# Ultraprecision Real-Time Displacements Calculation Algorithm for the Grating Interferometer System

**DOI:** 10.3390/s19102409

**Published:** 2019-05-27

**Authors:** Weinan Ye, Ming Zhang, Yu Zhu, Leijie Wang, Jinchun Hu, Xin Li, Chuxiong Hu

**Affiliations:** 1 State Key Laboratory of Tribology, Department of Mechanical Engineering, Tsinghua University, Beijing 100084, China; yewn12@mails.tsinghua.edu.cn (W.Y.); zhuyu@tsinghua.edu.cn (Y.Z.); wang-lj66@mail.tsinghua.edu.cn (L.W.); hujinchun@tsinghua.edu.cn (J.H.); lixin_09@mail.tsinghua.edu.cn (X.L.); cxhu@tsinghua.edu.cn (C.H.); 2 Beijing Lab of Precision/Ultra-Precision Manufacture Equipment and Control, Tsinghua University, Beijing 100084, China

**Keywords:** grating interferometer, ultraprecision, real-time, algorithm, rotation–translation coupling, geometric error

## Abstract

Grating interferometry is an environmentally stable displacement measurement technique that has significant potential for identifying the position of the wafer stage. A fast and precise algorithm is required for real-time calculation of six degrees-of-freedom (DOF) displacement using phase shifts of interference signals. Based on affine transformation, we analyze diffraction spot displacement and changes in the internal and external effective optical paths of the grating interferometer caused by the displacement of the wafer stage (DOWS); then, we establish a phase shift-DOWS model. To solve the DOWS in real time, we present a polynomial approximation algorithm that uses the frequency domain characteristics of nonlinearities to achieve model reduction. The presented algorithm is verified by experiment and ZEMAX simulation.

## 1. Introduction

Owing to its better environmental stability, an interferometric displacement measurement technique based on diffraction grating is an alternative to a laser interferometer for identifying the position of a wafer stage in a photolithography scanner [1,2,3]. For ultraprecision positioning of the wafer stage in a nonvacuum environment, the sensor and the displacements calculation algorithm are critical to the performance of the measurement system [4]. The algorithm is used to calculate the precise 6-DOF displacement of the wafer stage in real time using phase shifts and eliminate a rotation–translation coupling (similar to tilt-to-length coupling in a laser interferometer) and geometric errors, such as the Abbe and cosine errors [5,6,7].

For a multiple-DOF measurement system, the displacements calculation algorithm can be regarded as a method to solve unknown displacements by using known sensor readings. One simple approach is establishing a linear readings–displacements model using various approximations and deriving its closed-form solutions. Although this method has high resolution and excellent real-time performance, its calculation precision degrades with an increase in the rotation range because its nonlinearities such as rotation–translation coupling and geometric errors are neglected [8,9,10]. To achieve ultraprecise displacements calculation, establishing a precise readings–displacements model is a prerequisite. It is a common practice to use affine transformation to establish an exact functional relationship among displacements of the sensor and the measured object, and to consider the sensor reading as a linear function of the displacements of the sensor [11,12]. For a grating interferometer system, this modeling method ignores the nonlinear relationship between the phase shift and the displacements of the encoder (DOE) caused by rotation–translation coupling. Moreover, the model established by this method has no closed-form solution and can only be solved precisely by an iterative numerical algorithm, which has a large computational effort and cannot meet the requirements of real-time measurement [13]. In a similar study on the laser interferometer system, Gao et al. analyzed the changes of the external optical path of the laser interferometer caused by rotation using the above modeling method, and they established a precise model containing tilt-to-length coupling. The closed-form solution is derived after nonlinear model reduction using information from symmetrically arranged additional interferometers [4]. However, because absolute symmetry does not exist in actual measurement due to manufacturing and assembly errors, the method is still not precise enough. Ultraprecision requires a complex computational model, while real-time performance requires a simple model; therefore, it is challenging to achieve both goals simultaneously.

In this paper, we present an algorithm that can calculate the 6-DOF DOWS in a photolithography scanner in real time with ultraprecision. According to the two factors that cause the phase shift of the grating interferometer based on the Doppler effect—the diffraction spot displacements and the change in the internal and external effective optical paths—the exact relationship between the phase shift of each grating interferometer and the 6-DOF DOWS is established with affine transformation. To solve the DOWS in this complex model in real time, a polynomial approximation (PA) algorithm is presented in which model reduction is achieved by the frequency domain characteristics of the nonlinearities.

## 2. Readings-displacements Modeling Method

**Figure 1:**
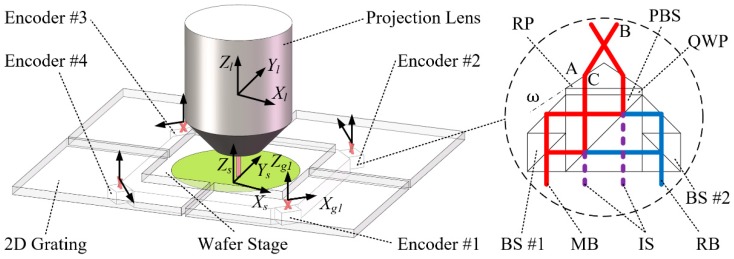
Four-grating interferometer system of the wafer stage and the concept of the encoder with Littrow diffraction for demonstrating the modeling method. Four two-dimensional gratings and the projection lens are installed in a metrology frame, and four 2-DOF encoders are mounted on the corners of the wafer stage. RP, Refraction Prism; QWP, Quarter Wave Plate; PBS, Polarizing Beam Splitter; BS, Beam Splitter; MB, Measuring Beams; RB, Reference Beams; IS, Interference Signals.

As shown in Figure 1, most advanced photolithography scanners adopt a planar motor drive system and a grating interferometer displacements measurement system [14]. The measurement system comprises four grating interferometers and is used to measure the 6-DOF DOWS relative to the projection lens. During the exposure process of the photolithography scanner, the wafer stage moves to some areas where only three encoders are active because another moves out of the area covered by the grating. Therefore, displacements calculation algorithms using both 4-active and 3-active encoder readings are necessary.

**Figure 2:**
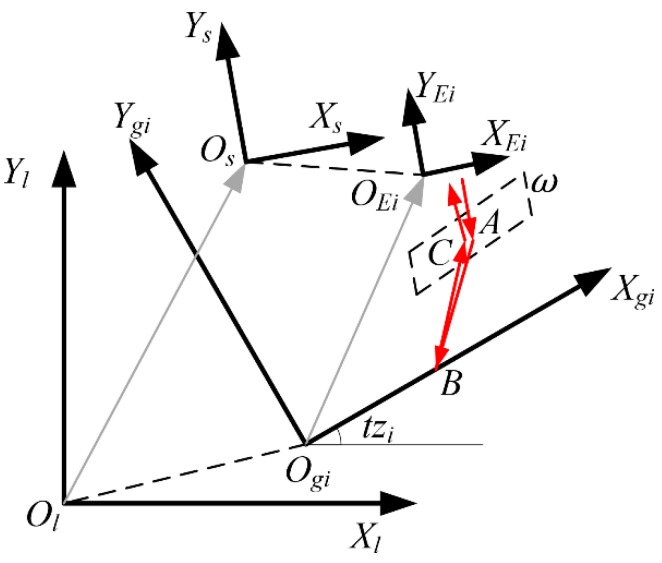
Definition of coordinate systems. The CS $O_{gi}X_{gi}Y_{gi}Z_{gi}$ fixed to the CS $O_{l}X_{l}Y_{l}Z_{l}$ represents the grating of the grating interferometer #i, and the CS $O_{Ei}X_{Ei}Y_{Ei}Z_{Ei}$ fixed to the CS $O_{s}X_{s}Y_{s}Z_{s}$ represents encoder #i. The Euler angles (z-y-x extrinsic) between the CS $O_{gi}X_{gi}Y_{gi}Z_{gi}$ and the CS $O_{l}X_{l}Y_{l}Z_{l}$ is $\left(tx_{i},ty_{i},tz_{i}\right)$. The red arrows represent the typical internal and external effective optical path of the encoder. Points A and C represent the refraction points of the measuring beams, and point B represent the diffraction spot.

To establish the precise phase shift-DOWS model, the grating interferometer system of the wafer stage shown in Figure 1 is reduced to some coordinate systems (CSs) and points, as shown in Figure 2. The 6-DOF DOWS CS $O_{s}X_{s}Y_{s}Z_{s}$ relative to the projection lens CS $O_{l}X_{l}Y_{l}Z_{l}$ is the measured DOWS $\left(Dx_{l},Dy_{l},Dz_{l},Rx_{l},Ry_{l},Rz_{l}\right)$. Points A, B, and C represent the key points in which the measuring beams change directions and are used to demonstrate the modeling method. When the relationship between the position of all key points and the DOWS is determined, the change ΔL of the total length of all measuring beams and diffraction spot displacements ΔS in the $X_{gi}$ direction can be calculated. Then, the phase shift φ of interference signal can be given by

(1) $$\varphi =-\frac{2\pi \alpha }{p_{x}}\Delta S+\frac{-2\pi n}{\lambda }\Delta L,$$

where α is the diffraction order, $p_{x}$ is the grating pitch in the $X_{gi}$ direction, n is the refractive index in the air, and λ is the wavelength of the laser.

The relationship between the DOE and ΔL, ΔS is more intuitive, and therefore, DOWS is first converted to the DOE in the CS $O_{gi}X_{gi}Y_{gi}Z_{gi}$. Rotational displacements can be transformed by the transformation matrix between the CS $O_{l}X_{l}Y_{l}Z_{l}$ and CS $O_{gi}X_{gi}Y_{gi}Z_{gi}$, and the translational displacements are transformed by Equation (2). When the DOE $\left(dx_{gi},dy_{gi},dz_{gi},rx_{gi},ry_{gi},rz_{gi}\right)$ is computed, the transformation from CS $O_{gi}X_{gi}Y_{gi}Z_{gi}$ to CS $O_{Ei}X_{Ei}Y_{Ei}Z_{Ei}$ can be represented by matrix QEigi. In the CS $O_{Ei}X_{Ei}Y_{Ei}Z_{Ei}$, the homogeneous matrix of point A that does not change with the DOE is a constant matrix AEi. The computation of the homogeneous matrix of point B requires the use of geometric constraints for beam AB: the direction vector DEiAB of beam AB can be calculated by the azimuth π and the Littrow angle $\theta_{l}$; the Z coordinate of point B in the CS $O_{gi}X_{gi}Y_{gi}Z_{gi}$ is 0. According to these constraints, Equation (3) is set to solve Bgi.

(2) Egi=[dxgidygidzgi1]T=Qgil−1QslEsiEsi=[mxsimysimzsi1]T, i=1,2⋯KQgil=[Rot(txi,tyi,tzi)[mxsimysimzsi]T0001]Qsl=[Rot(Rxl,Ryl,Rzl)[DxlDylDzl]T0001],

where Egi is a homogeneous matrix representing the translational DOE $\left(dx_{gi},dy_{gi},dz_{gi}\right)$ of the grating interferometer #i; Esi represents the position $\left(mx_{si},my_{si},mz_{si}\right)$ of encoder #i in the CS $O_{s}X_{s}Y_{s}Z_{s}$; Qgil is a transformation matrix representing the position and orientation of #i grating in the CS $O_{l}X_{l}Y_{l}Z_{l}$; $Rot\left(tx_{i},ty_{i},tz_{i}\right)$ is a rotation matrix corresponding to the $\left(tx_{i},ty_{i},tz_{i}\right)$ based on Euler angles (z-y-x extrinsic); Qsl represents the transformation matrix formed by the DOWS $\left(Dx_{l},Dy_{l},Dz_{l},Rx_{l},Ry_{l},Rz_{l}\right)$; and K is the number of active encoders.

(3) QEigi=[R3×3T3×10001]=[Rot(rxgi,rygi,rzgi)[dxgidygidzgi]T0001]BEi=AEi+[sinθlcosπsinθlsinπcosθl0]T|AB→|Bgi=QEigiBEi,bgi31=0,

where $\left|\vec{AB}\right|$ is the length of the beam AB, bgi31 is the Z coordinate of point B in the CS $O_{gi}X_{gi}Y_{gi}Z_{gi}$, and $\theta_{l}$ is the Littrow angle.

According to the above method, we establish the relationship between the position of all key points and the DOE. Then, ΔL(DOE) and ΔS(DOE) can be obtained, and the phase shift φ(DOE) is derived. Because φ(DOE) contains several trigonometric and inverse trigonometric functions, it cannot be expressed by an explicit function, and its computational efficiency is very low. Considering that the three rotational displacements are very small, φ(DOE) can be simplified to a polynomial with the Taylor series expansion at the point $\left(rx_{gi},ry_{gi},rz_{gi}\right)=\left(0,0,0\right)$, as shown in

(4) $$\varphi =\frac{2\pi dx_{gi}}{p_{x}}+u\left(rx_{gi},ry_{gi},rz_{gi}\right)dz_{gi}+v\left(rx_{gi},ry_{gi},rz_{gi}\right),$$

where u and v are the polynomials of $\left(rx_{gi},ry_{gi},rz_{gi}\right)$.

The order of Taylor’s expansion determines the precision of the polynomial’s reproduction of the original model, and the fourth-order Taylor expansion (cubic polynomial) can achieve an approximation precision on the order of picometers. In the practical application of the grating interferometer, the polynomial based on the design parameters cannot represent the precise nonlinearities owing to manufacturing and assembly errors. The polynomial calibrated by external sensors is more practical for the ultraprecision positioning of the wafer stage.

Equation (4) represents the phase shift-DOE model; however, the phase shift-DOWS model is required to calculate the DOWS. The DOE in Equation (4) is replaced by the DOWS by coordinate transformation, and the coefficients of polynomials are redetermined by the regression method. The phase shift-DOWS model of two interference signals $\varphi_{a}$ and $\varphi_{b}$ of the 2-DOF grating interferometer system in both 4-active and 3-active encoder modes can be derived as

(5) $$\begin{array}{l} \varphi_{ia}=f_{ia}\left(Dx_{l},Dy_{l},Dz_{l},Rx_{l},Ry_{l},Rz_{l},mx_{si},my_{si},mz_{si},tx_{i},ty_{i},tz_{i},C_{ia1},C_{ia2}\cdots \right)\\ \varphi_{ib}=f_{ib}\left(Dx_{l},Dy_{l},Dz_{l},Rx_{l},Ry_{l},Rz_{l},mx_{si},my_{si},mz_{si},tx_{i},ty_{i},tz_{i},C_{ib1},C_{ib2}\cdots \right) \end{array},$$

where $C_{i}$ is the redetermined coefficient of the polynomials.

## 3. Polynomial Approximation Algorithm with Substitution Variables

When the grating interferometer system reads out the phase shift, the DOWS can be calculated by solving Equation (5). However, due to the very complex nonlinearity, Equation (5) has only a numerical solution rather than a closed-form solution. To calculate the ultraprecision DOWS in real time, cubic polynomials of 6 or 8 normalized phase shift $\left(\varphi_{ia},\varphi_{ib}\right)$ are employed to approximate the calculation process of two measurement modes. Owing to the complex nonlinear coupling in the phase shift, a cubic polynomial of about 120 terms is required to ensure that the calculation errors of translational displacements are less than 10 pm in a non-ideally manufactured and assembled measurement system. Such a large polynomial requires considerable computational effort to be used for real-time measurement of 10 kHz or higher bandwidths.

**Figure 3:**
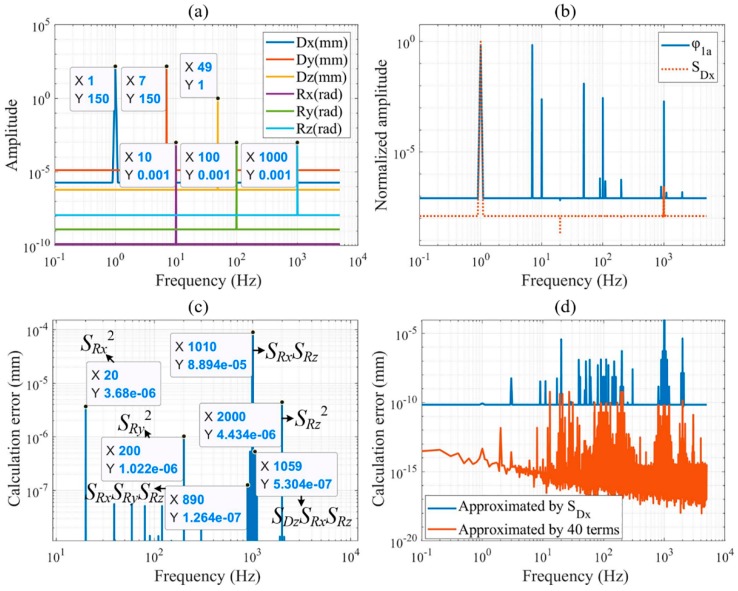
(**a**) Fourier amplitude spectrum of DOWS. (**b**) Fourier amplitude spectrum of normalized substitution variables and phase shift. (**c**) Fourier amplitude spectrum of the calculation error of $Dx_{l}$ with $S_{Dx}$. The basis functions to be added can be determined by the period of trigonometric functions of calculation errors. (**d**) Comparison of calculation errors between linear approximation and presented PA with substitution variables.

To further improve the real-time performance of the approximate model, a more efficient method for nonlinear model reduction is needed. The 6-DOF DOWS are set to sinusoidal motions at different frequencies, and the amplitude is set as the motion range of each DOF, as shown in Figure 3a. As shown in Figure 3b, six substitution variables $\left(S_{Dx},S_{Dy},S_{Dz},S_{Rx},S_{Ry},S_{Rz}\right)$, which are only dependent with each displacement by linear fitting the normalized phase shift, are established by Equation (6). When each displacement is calculated using only the corresponding quasi-linear substitution variable, the frequency domain characteristics of the displacement calculation error are shown in Figure 3c. The peak in the Fourier amplitude spectrum shows the magnitude and frequency of the error, which can be eliminated by adding a basis function corresponding to the error frequency to the PA model. By adding basis functions in the order of error amplitude from large to small until the precision requirement of the displacement calculation is satisfied, the PA model with the highest computational efficiency is established. As shown in Equation (7), the displacements calculation algorithm obtained by this method only requires 40 terms to ensure that the calculation errors of translational and rotational displacements are less than 10 pm and 1 nrad, respectively.

(6) $$\left[\begin{array}{cccccc} S_{Dx} & S_{Dy} & S_{Dz} & S_{Rx} & S_{Ry} & S_{Rz} \end{array}\right]=\left[\begin{array}{ccccc} \varphi_{1a} & \varphi_{1b} & \cdots & \varphi_{ia} & \varphi_{ib} \end{array}\right]C1_{2K\times 6},$$

(7) $$DOWS_{6\times 1}=C2_{6\times 40}{\left[\begin{array}{cccccccccc} S_{Dx} & S_{Dy} & S_{Dz} & S_{Rx} & S_{Ry} & S_{Rz} & S_{Rx}S_{Rz} & S_{Rx}{}^{2} & S_{Ry}{}^{2} & \cdots \end{array}\right]}_{40\times 1},$$

where $C1_{K\times 6}$ is the linear approximation coefficient matrix of the normalized phase shift to the DOWS and $C2_{6\times 40}$ is the coefficient matrix of the PA algorithm consisting of 40 basis functions of six substitution variables.

## 4. Experiment and ZEMAX Simulation

### 4.1. Experiment

In actual measurement, the precision of the presented PA algorithm driven by data of analytical model depends on two key factors. One is the precision of geometric parameters in the phase shift-DOWS model, the calibration of which will be carried out as future work. The other is the ability of algorithm to reproduce the precision of analytical model under actual measurement noise, which is verified by the following simplified experiment instead of the extremely complex and costly system as shown in Figure 1.

**Figure 4:**
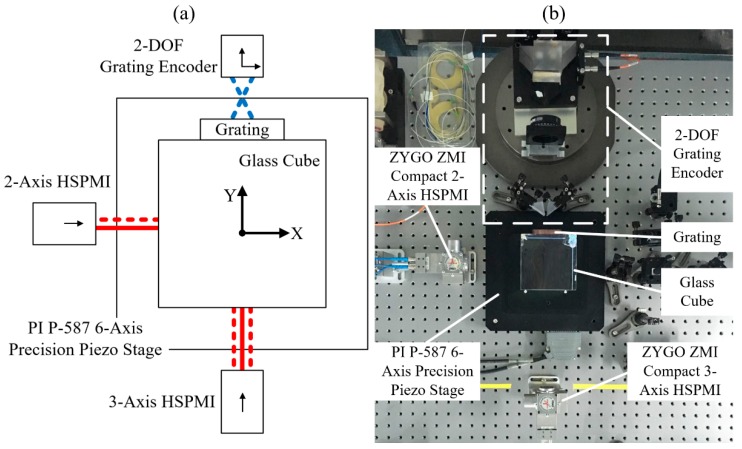
(**a**) 5-DOF experimental scheme. (**b**) 5-DOF experimental device. HSPMI is the commercial laser interferometer. Red lines indicate commercial laser interferometer system, and dotted lines indicate hybrid interferometer system.

**Figure 5:**
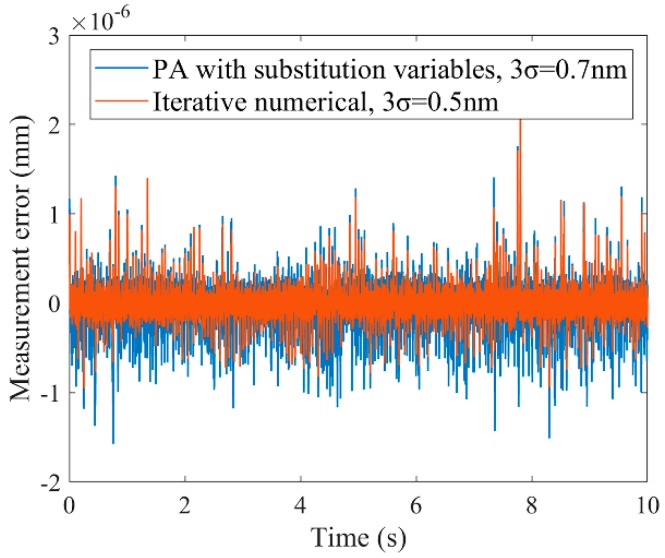
Measurement errors of the iterative numerical algorithm and the presented PA algorithm in the X direction.

Since the measuring principle is similar to that of the grating interferometer, a laser interferometer system can also use the proposed modeling and calculation methods. Therefore, we set up an experimental device with a commercial laser interferometer system and a self-made grating interferometer, as shown in Figure 4a,b. A more detailed description of the experimental device can be found in our previous research [15]. In ZEMAX (ZEMAX is software for optical system design) simulation, when the rotational displacements of the Precision Piezo Stage were within the range of ±0.05 mrad, the tilt-to-length coupling of the commercial laser interferometer system is less than 100 pm and can be ignored. The readings of commercial laser interferometer system can be regarded as linear functions of translational displacements, and therefore, the displacement calculation model of commercial laser interferometer system can be established according to Equation (2). The phase shift-DOE model of the self-made grating interferometer can be calibrated with the measurement data of commercial laser interferometer system, and the displacement calculation model of the hybrid interferometer system can be established. In the experiment, the Precision Piezo Stage performs a sinusoidal motion with an amplitude of 5 μm in the *X*-axis and a sinusoidal rotation with an amplitude of 10 μrad around the *Z*-axis simultaneously. Then, the iterative numerical algorithm and the presented PA algorithm are used to calculate the displacements of the Precision Piezo Stage with the readings of the hybrid interferometer system. Considering the measurement result of the commercial laser interferometer system as the actual value, the measurement errors of the hybrid interferometer system are shown in Figure 5. Since the iterative numerical algorithm can represent the precision of the analytical model, the results prove that the presented PA algorithm can reproduce the precision of analytical model under actual measurement noise in a system containing grating interferometers.

### 4.2. ZEMAX Simulation

**Figure 6:**
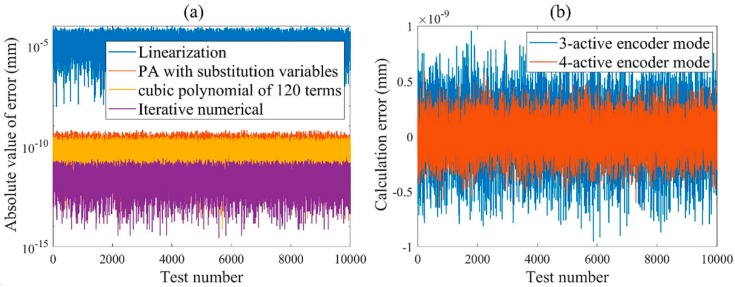
(**a**) Comparison of the absolute values of the calculation errors of the four algorithms. (**b**) Comparison of the calculation errors when using the presented algorithm for the 4-active encoder mode and the 3-active encoder mode.

Owing to its picometer-scale calculation precision is far less than the measurement noise of the commercial laser interferometer system and self-made grating interferometer, the performance of the presented PA algorithm cannot be verified by the experiment. To fully verify the performance of the proposed modeling and calculation method, the simulation model of the nonideally manufactured and assembled grating interferometer system in Figure 1 is built in the nonsequential mode of ZEMAX. First, a series of 6-DOF DOWS are randomly generated in their maximum range, and the phase shift is calculated by the simulation model and the presented analytical model. The error of the analytical model compared to the simulation model is on the order of 10^−9^ rad, and the corresponding displacement error is on the order of 10^−5^ pm. Then, based on the analytical model of the 4-active encoder mode, 6-DOF DOWS as shown in Figure 3a are generated to establish the substitution variables in Equation (6) and the displacements calculation algorithm in Equation (7). Finally, the phase shift corresponding to 10000 sets of random DOWS within the range shown in Figure 3a obtained by simulation are substituted into the four algorithms of linearization, iterative numerical, cubic polynomial of 120 terms, and PA with substitution variables to calculate the DOWS; the displacement calculation errors are shown in Figure 6a. The performance comparison of four algorithms is summarized in Table 1, in which the time is obtained from the calculation in MATLAB with an Intel Core i7-8700K CPU @ 3.70 GHz. When the proposed algorithm is applied to the 3-active encoder mode, the calculation error remains less than 10 pm, as shown in Figure 6b.

## 5. Conclusions

We presented an algorithm to calculate the 6-DOF DOWS in a photolithography scanner in real time with ultraprecision. We established the exact relationship between the phase shift of each grating interferometer and the 6-DOF DOWS based on affine transformation considering the two factors that cause the phase shift of the grating interferometer based on the Doppler effect. To solve the DOWS in real time, we presented a PA algorithm in which the frequency domain characteristics of the nonlinearities are used to achieve model reduction. The experimental results verified the ability of the proposed PA algorithm to reproduce the precision of analytical model under actual measurement noise, and the simulation results showed its performance, taking only 1.7 μs to achieve calculation errors of translational displacements less than 10 pm.

## Figures and Tables

**Table 1:** Performance comparison of the four algorithms.

Algorithm	Calculation Error		Calculation Time
	Translational	Rotational	
Linearization	8.4 × 10^4^ pm	11.3 nrad	1.3 μs
PA with substitution variables	1.8 pm	0.6 nrad	1.7 μs
Cubic polynomial of 120 terms	0.3 pm	9.7 × 10^−4^ nrad	13.8 μs
Iterative numerical	0.03 pm	2.8 × 10^−4^ nrad	67.4 μs
